# Assessment of the impact of unplanned extubation on ICU patient outcome

**DOI:** 10.1186/cc9589

**Published:** 2011-03-11

**Authors:** E Bastos de Moura, J Aires de Araújo Neto, M De Oliveira Maia, F Beserra Lima, R Fernandes Bomfim

**Affiliations:** 1 Hospital Santa Luzia, Brasília, Brazil

## Introduction

The objective of this study is to investigate and analyze the events of unplanned extubation (UE) in the ICU of Santa Luzia Hospital, Brasília, Brazil. Incidence rates of unplanned extubation vary; reported rates range from 3% to 14%. This phenomenon occurs during procedures performed by healthcare workers, or in self-extubation if the patient removes the endotracheal tube. Unplanned extubations are considered an indicator of healthcare quality in the ICU. Reintubation may be necessary and is associated with complications, including emergency cricothyrotomy, cardiac arrest, and death.

## Methods

A retrospective cohort study, analysing the cases of UE reported between January 2009 and June 2010 in Santa Luzia Hospital's ICU. In this period 3,302 patients were admitted, and 551 were submitted to mechanical ventilation (MV). The cases of UE are notified through proper form by the physiotherapy. The incidence rate of unplanned is calculated by the relationship between the number of patients extubated accidentally and the number of patients intubated/day, multiplied by 100.

## Results

The incidence rate of UE was 0.21% (nine patients in 4,232 days of MV). Only two extubations (22.22%) occurred accidentally while seven cases (77.78%) were self-extubation. Patients were predominantly female (55.56%; *n *= 5), mean age was 59.86 ± 27.28 years, mean SAPS II score of 35.33 ± 12.50 (RISK: 21.56 ± 18.32%), mean APACHE II score of 10.44 ± 6.27 (RISK: 17.11 ± 15.35%), mean duration of MV 8.68 ± 9.81 days, mean length of stay in ICU 15.89 ± 8.75 days. Two patients (22.22%) needed reintubation. In only one patient (11.11%) urgent cricothyrotomy was required due to difficulty on reintubation. Most patients had already started the weaning process (77.78%). The leading cause of accidental extubation was failure of restraint (88.89%) associated with psychomotor agitation (55.56%). We had three (33.33%) cases of death in the group, but not associated with the UE.

## Conclusions

In the studied population we observed a low incidence of this adverse event, which demonstrates effectiveness in prevention strategies adopted. Reintubation and urgent cricothyrotomy rates were low, which resulted in increased length of stay in the ICU and MV.
